# Who watches the worms? Motivation and (non-)participation in a contributory citizen science project

**DOI:** 10.1186/s12862-025-02471-y

**Published:** 2025-12-24

**Authors:** Victoria J. Burton, Alan G. Jones, Lucy D. Robinson, Paul Eggleton, Andy Purvis

**Affiliations:** 1 Natural History Museum, London, SW7 5BD UK; 2 Science and Solutions for a Changing Planet DTP, Department of Life Sciences, Imperial College London, London, SW7 2AZ UK; 3 Earthwatch, Mayfield House, 256 Banbury Road, Oxford, OX2 7DE UK; 4 Department of Life Sciences, Imperial College London, Silwood Park, London, SL5 7PY UK; 5 Scion(New Zealand Forest Research Institute), Tītokorangi Drive, Private Bag 3020, Rotorua, 3046 New Zealand

**Keywords:** Citizen science, Community science, Soil biodiversity, Earthworms, Participant motivation

## Abstract

**Background:**

Citizen science projects rely on public participation to generate data and promote engagement with science. However, little is known about the motivations of individuals who register for citizen science projects but ultimately do not participate. Understanding non-participation is important for improving recruitment and engagement strategies. This study used *Earthworm Watch*, a UK-based soil biodiversity citizen science project that ran from April 2016 to August 2018, to explore the motivations of both participants and non-participants, and to examine how these relate to demographic factors and survey completion rates.

**Results:**

A total of 1,678 participants registered for *Earthworm Watch*. The overall survey return rate was 12.75%, with no significant differences by age or gender. The provision of physical survey packs did not significantly affect completion rates. Direct contact with project staff was the only recruitment method associated with a significantly higher survey return rate. Significantly more registrants were female than male across all age groups. Motivations related to *understanding* and *values* were most reported, with participants often expressing a desire to learn more or to contribute to the topic, but these participants contributed fewer surveys than those without those motivations. *Social* motivations were mentioned less frequently but were more commonly reported by women. Younger participants were more likely to cite *career*-related motivations.

**Conclusions:**

The limited impact of physical materials on participation suggests that designing projects for immediate and accessible involvement could be more cost-effective. The significant influence of meeting project members and hands-on experiences at events strengthens the case for including these activities in engagement plans. Motivations to participate in *Earthworm Watch* varied by demographic factors such as age and gender; however, when significant, they influenced only the number of surveys submitted, not the likelihood of participation. This indicates that a one-size-fits-all approach to engagement may be ineffective. Tailoring recruitment and feedback strategies to align with participant demographics and motivations may enhance engagement and survey return rates in future citizen science projects.

**Supplementary information:**

The online version contains supplementary material available at 10.1186/s12862-025-02471-y.

## Background

The benefits of citizen science, also known as community science, are numerous, both scientific and social, including collecting data for environmental monitoring and decision-making at the local [1], national [2], and international [3] scales. Benefits for participants include gaining knowledge [4], improving employment prospects [5] and enhancing well-being and connection to nature [6]. However, for these benefits to be realised, volunteers must be successfully recruited and retained, which can be a challenge [7]. Studies on the motivations and recruitment of participants are less published than other areas of citizen science [8, 9] but given its importance is a growing area of research [10, 11].

One widely used model for understanding volunteer motivations is the Volunteer Functions Inventory (VFI) [12], which identifies six motivational categories: *values* (altruistic or humanitarian), *career* (career-related benefits), *enhancement* (self-improvement), *understanding* (learning new things), *social* (meeting new people or socialising in general), and *protective* (addressing personal problems or reducing negative feelings). Another common distinction is between intrinsic motivations (inherently interesting or value-aligned) and extrinsic motivations (achieving an external goal) [13]. Within citizen science, participation is often driven by intrinsic values, such as a desire to help wildlife or contribute to scientific knowledge - although the latter could arguably be considered extrinsic if framed as an external goal. Motivational patterns also vary across age and socioeconomic groups [11] and over the lifespan of a project [14, 15]. A common limitation of existing studies is their focus on the motivations of participants who have completed projects or remain active, leaving the motivations of individuals who registered but did not participate, or who withdrew early, under-researched [11, 16, 17]. Although other frameworks have been applied to citizen science, for example, Batson, Ahmad and Tsang [18] employ a four-category model of egoism, altruism, collectivism, and principlism - the VFI remains the most widely used in citizen science and environmental volunteering research [19]. This is despite critiques that it is neither exhaustive nor exclusive, and that most motivations contain both intrinsic and extrinsic elements [20]. To enable comparability, we use the VFI in our study despite its limitations.

The subject of this case study, *Earthworm Watch* [21], was a contributory citizen science project, following Bonney’s [22], definition, which ran from April 2016 to August 2018. The project aimed to improve understanding of earthworm abundance and community composition in urban areas. Participants registered through the project website and could either request a survey pack by post or download instructions and use their own materials. Each survey pack contained a booklet with instructions and a data entry form, a chart for identifying earthworm groups and soil types, two sachets of malt vinegar for testing soil alkalinity, two 15 g bags of mustard powder to extract earthworms, and a pair of nitrile gloves.

The survey took about an hour and involved sampling two 20 cm × 20 cm areas located a few metres apart in contrasting habitats or management conditions. In each area, participants recorded habitat characteristics, then dug out, counted, and identified earthworms into three categories, and measured basic soil properties. Results were uploaded via the project website, where participants could view their submission on a map, or alternatively posted to the organisers. Updates on scientific findings were shared through the website, e-newsletter, and social media channels. At registration, participants were asked why they wished to take part, providing an opportunity to record the motivations of both participants and non-participants.

Data on participant characteristics and motivations can reveal patterns in registration and survey completion, identify which participants are most likely to complete the survey, and assess whether the choice of downloading materials or receiving a physical pack influenced completion rates. Because physical packs were resource-intensive, it was important to evaluate whether they improved completion compared to downloadable materials. From experience, two opposing factors were considered: downloading materials offered immediacy but required participants to source mustard powder and vinegar themselves, whereas physical packs provided these items but introduced a delivery delay that could reduce engagement. This study therefore addresses the following hypothesis and research questions:

- Hypothesis: Survey completion is most likely to occur shortly after registration, with a rapid decline over time as interest wanes or physical packs are misplaced.
- Which aspects of project design and participant motivations are associated with higher participation?
- Which VFI motivations are most frequently expressed by participants at registration?
- Do motivations differ by age group and gender?

## Methods

*Earthworm Watch* ran from April 2016 to August 2018, with participants registering via a project website. During registration, they were asked voluntary questions on their gender, age bracket, and free-text boxes on how they heard about the project, and their reasons for wanting to take part.

Participant registration and survey completion data were obtained from the project website and used to calculate basic descriptive statistics on participant characteristics. One registrant declined to provide gender information, and 26 declined to provide age; these cases were excluded from statistical analyses due to small sample sizes. Registrants’ responses to the question “*Where did you hear about the project*?” were coded into eleven categories based on their free-text answers (Table 1). Blank or unclear responses were left uncoded.

**Table 1:** Categories used to code responses to the question “*Where did you hear about the project*?” based on registrants’ free-text answers. Blank or unclear responses were left uncoded

Category	Definition
Digital newsletter	Email newsletters sent by project partners or affiliates.
Event	Conferences, talks, or public events where the project was promoted.
Newspaper	Articles in print or online newspapers.
Online search	Discovery through search engines (e.g., Google).
Magazine	Print or digital magazine articles.
Project staff	Direct communication from project team members.
Radio	Mentions on radio programmes.
Social media	Posts or shares on platforms such as Facebook or Twitter.
Television	Mentions on TV programmes or news.
Website	Information found on the project’s official or partner websites.
Word of mouth	Personal recommendations from friends, family, or colleagues.

Responses to the question “*Why would you like to take part?*” were thematically coded in Dedoose [23] using the Volunteer Functions Inventory (VFI) [12] into the motivations *career*, *enhancement*, *protective*, *social*, *understanding*, and *values*. Text that was blank or lacked meaningful content was left uncoded. Because some responses reflected multiple motivations, coding was binary (0/1), allowing a single response to score in several categories.

## Survival analysis

Survival analysis was applied to test our hypothesis on the time between registration and survey submission, and to assess whether this interval varied by participant characteristics or by the method of obtaining survey materials (physical pack versus download). Each registrant was coded 1 or 0 whether they completed the survey or not, and for those who did, the time between registration and survey submission was calculated. There were 25 registrants with inaccurate registration dates (appearing to have registered after they had completed the survey) who were removed from analysis. Survival analysis was carried out with the package *survival* [24] in R version 4.4.2 [25].

To visualise the relationship between the time lag from registration to submission and whether the survey was completed, we constructed a Kaplan-Meier curve [26, 27]. We investigated the effect of participants’ age, gender and method of obtaining the survey pack (download or physical) on the probability that the survey would not be completed by fitting a Cox proportional hazards regression model [28].

We fitted a further Cox proportional hazards regression model to test for differences between different ways that the participants heard about *Earthworm Watch*, after first removing 206 data points where this was not coded. Due to small sample sizes, the *newspaper* category (*n* = 54) was combined with *magazines* (*n* = 103), and the *project staff* category (*n* = 36) was combined with *events* (*n* = 187) for analysis. The *radio* (*n* = 35) and *television* (*n* = 10) categories were excluded.

Because there are likely to be temporal biases when participants signed up to the survey (e.g., due to project promotion, weather conditions and school holidays), for both models, each participant’s registration time since the project start was included in addition to the time to completion. Models were plotted using *survminer* [29].

## Modelling

Analysis took place in R version 4.4.2 [25]. Due to having a single sample, the *protective* motivation was removed before analysis. The data were first checked for multicollinearity by calculating variance inflation factors (VIFs), all of which were below 5, indicating acceptable levels. To test whether different motivations for participating in *Earthworm Watch* influenced the number of surveys submitted, we modelled the count of submitted surveys as the response variable and the five remaining motivations (*Career*, *Enhancement*, *Social*, *Understanding*, *Values*) indicators as predictors. Because of the high proportion of zero counts, and strong overdispersion, we fitted a hurdle negative binomial model using the *glmmTMB* package [30, 31]. The hurdle model consists of two components: (1) a binomial logistic part modelling the probability of not submitting a survey, and (2) a truncated negative binomial part modelling the number of surveys among participants who submitted at least one. Both components included the same set of predictors.

To test whether motivations for participating in *Earthworm Watch* varied significantly by age group or gender, a series of multivariate logistic regression models was fitted, with each motivation modelled separately with age category and gender. Age 35–44 females were used as a baseline as this group were by far the largest registered (*n* = 295). Due to low frequencies for the *career* motivation, age categories 45 and above were combined before modelling. Plots for models were generated using the *ggplot2* package [32].

## Cluster analysis

Due to low frequencies, the motivations *protective* and *career* were excluded from the cluster analysis. We used hierarchical cluster analysis using Ward’s method [33] to determine whether motivations were independent or tended to co-occur. Age group and number of surveys were normalised to a 0 – 1 scale, as hierarchical clustering with Ward’s method is sensitive to differences in variable scales. The data were first checked for multicollinearity by calculating variance inflation factors (VIFs), all of which were below 5, indicating acceptable levels. The analysis was performed using the *cluster* package [34] in R version 4.4.2 [25]. The *NbClust* package [35] was used to help identify the optimal number of clusters. Finally, the age and gender of the participants in each cluster were examined to assess whether motivational profiles were associated with specific demographic characteristics.

## Results

A total of 1,678 participants registered for *Earthworm Watch*, 214 of whom completed at least one survey - a return rate of 12.75%. In total, 334 surveys (two soil pits each) were completed, as well as 13 incomplete surveys that submitted data from only one soil pit.

**Fig. 1:**
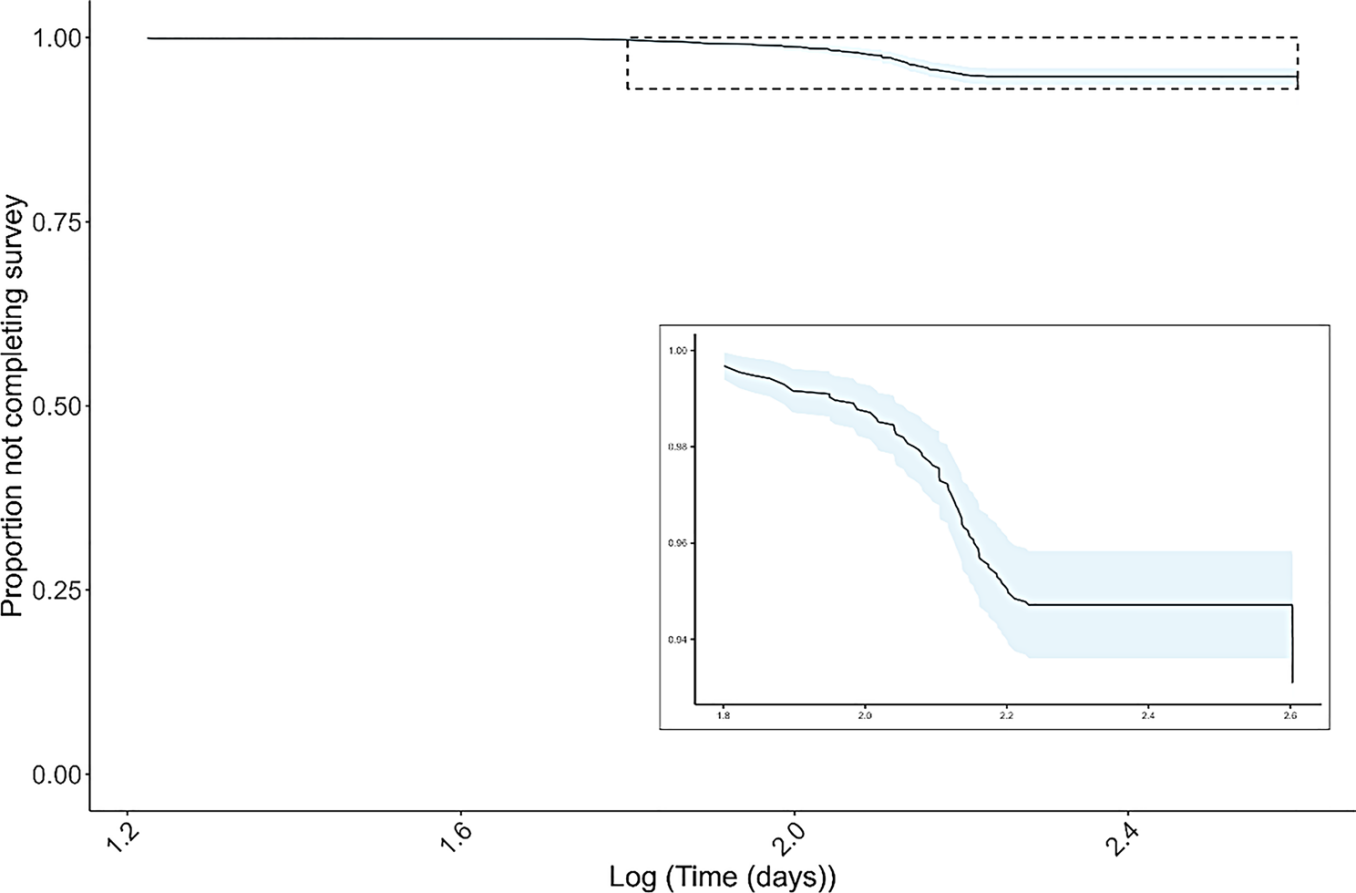
Kaplan-Meier curve showing the proportion of *Earthworm Watch* participants completing their survey over time. Shaded areas show 95% confidence intervals. The main plot shows the full range of data, while the inset shows the region of the y-axis between 0.93 to 1.0

**Fig. 2:**
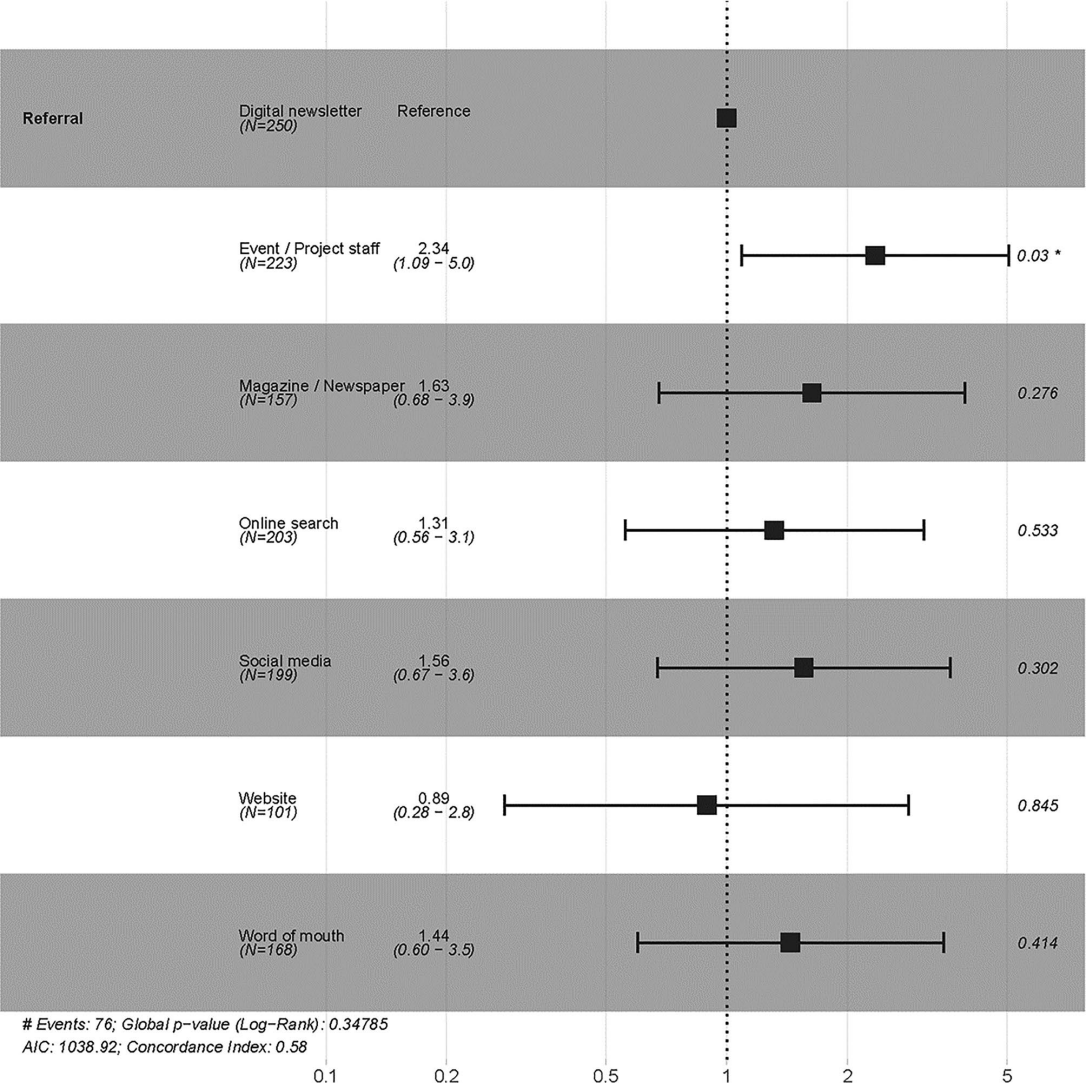
Summary of the Cox proportional hazards regression model for the likelihood of *Earthworm Watch* survey returns by referral category, for participants who reported where they heard about the survey. Hazard ratios (HR) with 95% confidence intervals (CI) are shown for each category. An hr > 1 indicates an increased likelihood of survey return, while an hr < 1 indicates a decreased likelihood, compared to the reference category (*digital newsletter*). The vertical line at hr = 1 represents no effect. N denotes the number of participants in each category. p-values are displayed on the right; stars indicate significance (*p* < 0.05)

The proportion of participants completing the survey was not constant over time (Fig. 1) but levelled, suggesting that completion of the survey is unlikely after a certain period. Return rates varied among recruitment routes, but events and contact with project staff had significantly higher survey completion rates (*p* = 0.04, Fig. 2). There was no significant difference in return rate between participants who received a physical pack and those who downloaded a pack and sourced their own materials, or with age or gender (Figure S1).

**Fig. 3:**
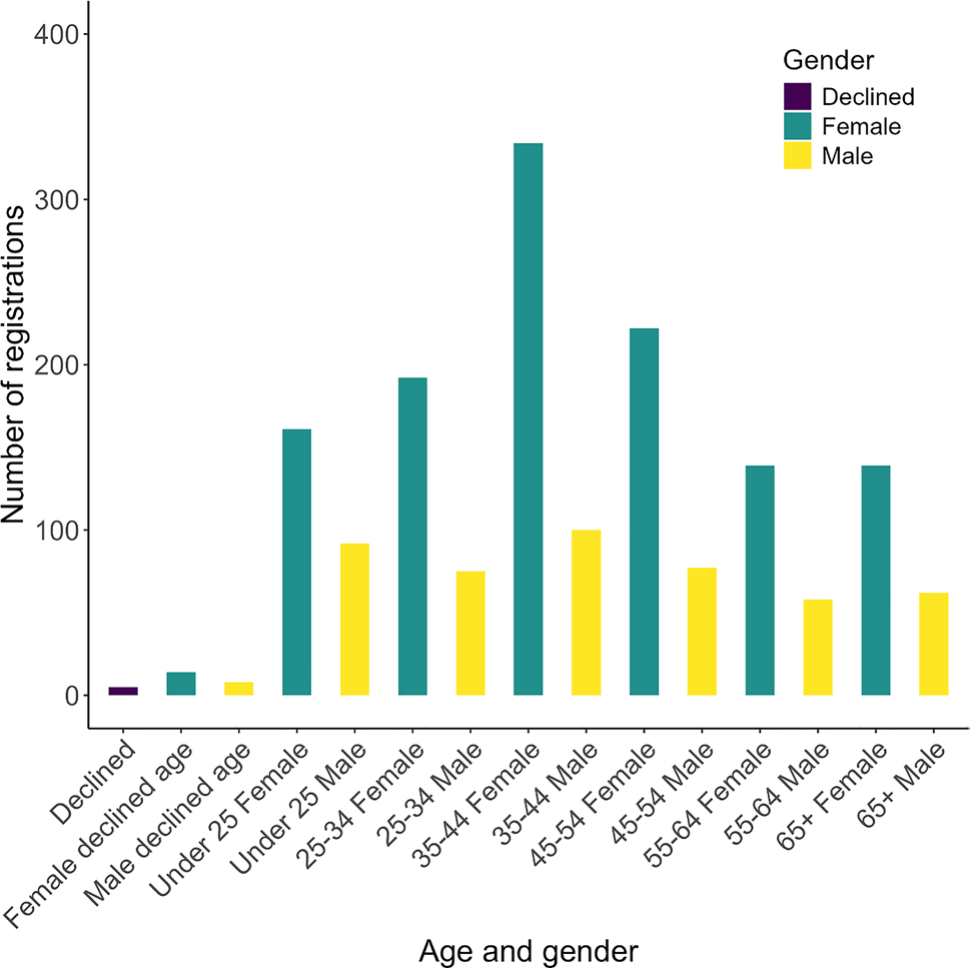
Age and gender distribution for registered *Earthworm Watch* participants

Significantly more registrants reported as female than male (472 males out of 1,201 registrants; exact binomial test, *p* < 0.001, observed proportion = 0.393, 95% CI: 0.365–0.421). This gender imbalance was evident across age classes (Fig. 3).

After removing uncoded samples, the motivations of 1,424 *Earthworm Watch* registrants were analysed. The most common were *understanding* motivations (842 responses, 59.1%), for example, “*I am running a STEM club at a primary school and we would love to be involved in some real scientific studies*”, followed by *values* motivations (609, 42.8%): “*Worried about wildlife and want to make a difference*”. There were smaller numbers of *enhancement* (164, 11.5%) and *social* (137, 9.6%) motivations, both of which are reflected in responses such as: “*I love wildlife and conservation, this sounds a fun activity to do with my kids, who love wildlife, but find school science “boring”*. The *career* category had the second fewest responses (33, 2.3%): “*I am currently studying towards a degree in Environmental Management and although the theoretical aspect of sampling has been discussed, we did not do any practical project. It would be interesting to get a feel of that and see how the results would be analysed and used.*”. There was only a single *protective* motivation (0.1%).

In the count component of the hurdle negative binomial model, two motivations significantly influenced the number of surveys completed. Participants motivated by *understanding* had an IRR of 0.62 (95% CI: 0.44–0.87, *p* = 0.006), and those motivated by *values* had an IRR of 0.63 (95% CI: 0.45–0.87, *p* = 0.005), indicating fewer surveys submitted compared to participants not motivated by these factors. In contrast, in the hurdle (zero) component, no motivations were associated with the likelihood of submitting zero surveys (all *p* > 0.05). These results suggest that, where significant, motivations influence the intensity of participation rather than the likelihood of any participation.

**Fig. 4:**
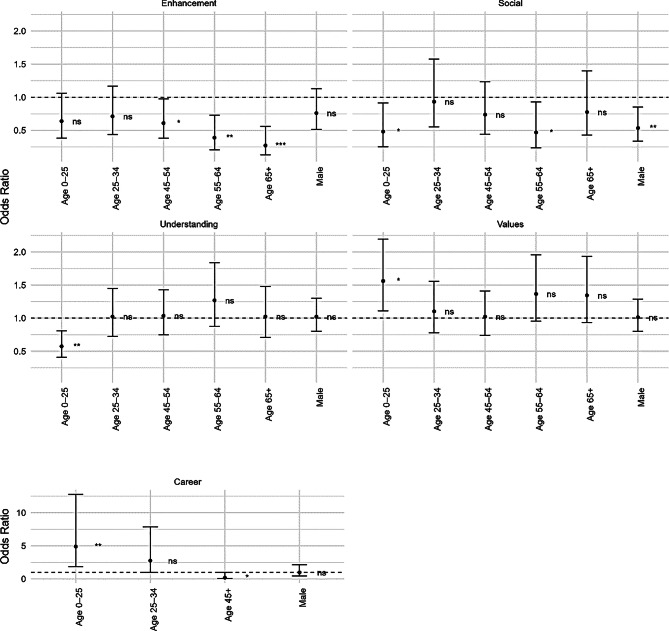
Odds ratios and 95% confidence intervals showing the effect of age group and gender on reported motivation for taking part in Earthworm Watch. Each point represents the estimated odds ratio relative to the reference group of age 35-44 females. Asterisks indicate the level of significance: <0.05*, <0.01**, <0.001*** ns = not significant

Only the *social* motivation showed a gender effect, with males having significantly lower odds of reporting this motivation than females (OR = 0.54, *p* = 0.009, Fig. 4). The *enhancement* motivation showed a relationship with age, with age groups 45 and above statistically less likely to report this motivation for signing up compared to the reference age group (35–44), the effect becoming more significant with age. Compared to the reference group, *social* motivations for taking part were reported less by those aged 0–25 (OR = 0.48, *p* = 0.025, Fig. 3) and older adults aged 55–64 (OR = 0.47, *p* = 0.030, Fig. 3). Younger (aged 0–25) registrants also had significantly lower odds of *understanding* motivations (OR = 0.57, *p* = 0.0014, Fig. 3). However, the youngest group had significantly higher odds of displaying *values* motivations for joining the project (OR = 1.56, *p* = 0.01, Fig. 3); other age groups showed no significant differences. Compared to the baseline, the youngest group were much more likely (OD = 4.9, *p* = 0.0012, Fig. 3) to mention *career* motivations when signing up for *Earthworm Watch*, and the oldest group (45+) had significantly lower odds (OD = 0.20, *p* = 0.05, Fig. 4), with only two examples in this age category however small sample sizes mean these findings should be interpreted with caution.

Using the *NbClust* package, the majority rule indicated three clusters (Figure S2) as the optimal solution. However, the quality of the clustering was low, as indicated by a modest average silhouette width (mean = 0.29). Silhouette values varied across clusters, with Cluster 1 (589 participants) showing moderate cohesion (average silhouette = 0.43), Cluster 2 (302 participants) and Cluster 3 (533 participants) having low silhouette values (0.02 and 0.28, respectively), indicating poor separation between groups (Figure S3). These results suggest that the clusters are not strongly distinct and should be interpreted with caution.

## Discussion

Few participants completed the survey immediately after registering, which may reflect delays in receiving posted packs. In line with our hypothesis, completion rates increased over time but soon plateaued, suggesting that participants were unlikely to complete the survey beyond a certain point. This may be due to loss of interest, misplacing the pack, or assuming the data was no longer needed. For future surveys, it could be valuable to test whether implementing a time limit on survey completion improves response rates.

Participants who received a physical pack were no more likely to complete the survey than those who did not. This was disappointing given the cost and effort involved in producing the packs, but it supports the idea that enabling immediate participation may increase engagement. It also suggests that citizen science projects should, where possible, be designed to require only commonly available household materials to maximise completion. Age or gender had no significant effect on survey completion.

Return rates varied by recruitment route, with the highest rates among participants who learned about *Earthworm Watch* through digital newspapers or magazines. Disappointingly, given the effort required for the team to appear on TV and radio programmes, these channels yielded comparatively low recruitment numbers. This pattern has also been observed by other researchers in ecology-focused citizen science projects [36], which may suggest that broadcast media is less effective for driving participation than more targeted approaches, such as direct engagement through social media, digital newsletters, and contact with team members. In our study, in-person engagement by project scientists and team members, either through events or directly, was the only recruitment method associated with a significantly higher survey completion rate.

Consistent with findings from other science and environmental citizen science projects and volunteering programmes [17], the most reported motivation for participating in *Earthworm Watch* was a desire to increase knowledge and *understanding* of the topic. *Values*-based motivations were also frequently reported, in keeping with previous work [11, 37]. For example, registrants often mentioned wanting to “help science,” “help the environment,” or support earthworms or a specific site. However, very few explicitly referred to helping the organisations running the project by name. These motivations were also the only two that showed significant effects on survey completion, with participants motivated by them submitting fewer surveys than others. One possible explanation is that completing a single survey was enough to meet their goals. While participants could take part multiple times, the project did not explicitly encourage repeat participation.

The frequency of *values* and *understanding* motivations, along with 1,678 registrations for *Earthworm Watch* over two years, indicates that although earthworms receive less attention in citizen science compared to other taxa such as birds and butterflies, there is still clear public interest. This is supported by studies showing that the public generally perceives earthworms positively, such as “useful for the soil” [38]. Earthworms are also among the most frequently studied soil biodiversity groups in citizen science, with at least 20 European projects identified in a forthcoming review (Barantal et al., accepted). The relatively low percentage of data submissions may reflect barriers to participation rather than poor perception of the group, such as the inconvenience of digging or reluctance to submit data. Alternative protocols could be considered, such as timed searches in microhabitats under pots or logs, though this would bias toward certain ecological groups, or timed counts of earthworm casts, which measure activity rather than abundance, as substitutes for soil pit sampling.

*Social* motivations were mentioned less frequently than *understanding-* and *values*-based motivations; they were the only category to show a significant difference between genders, with women reporting more social motivations for participating than men. This is consistent with findings by West et al. [11] who found that studies with more than 50% women in their sample rated social motivations higher than studies with fewer women, and other studies [39]. *Earthworm Watch* had a clear female bias in registration across all age categories. Some citizen science projects and reviews have found female bias [40, 41], although a self-reported survey of participants found an event split [42], and Pateman et al. [43] found that more men than women have participated in citizen science in the UK. Evidence from bird-related citizen science and leisure activities [44] suggests women prefer altruistic projects. *Earthworm Watch*, promoted as contributing to science and improving soil health, would be classified as altruistic, so the female bias in registration is consistent with this hypothesis. However, a limitation of our study is that we recorded only the gender of those who registered for the survey, not those who completed it. It is possible that mothers were more likely than fathers to register their children for *Earthworm Watch*, which is consistent with the findings by Castell et al. [45] that women play a key role in informal science learning and are more likely than men to accompany participants to science-related leisure activities.

As in West et al. [11], and in line with suggestions by other authors [12] we found that younger citizen scientists were more likely to report motivations related to personal development. Participants under the age of 25 were significantly more likely to mention career-related motivations. This suggests that if organisations wish to engage this generation, often described as less connected to nature [46], emphasising skill development and offering recognition for it may support recruitment and retention. This age group expressed more *values*-based than *understanding*-based motivations compared to the reference group, which contrasts with other studies that found *values*-based motivations to be more common among older volunteers [11]. This finding challenges the assumption that *values*-based motivations are more associated with older volunteers. This finding also has implications for addressing eco-anxiety among younger demographics. Values-based motivations, such as contributing to environmental protection, may reflect a desire to take meaningful action in response to climate and biodiversity crises. Citizen science projects can provide a constructive outlet for these concerns by enabling participants to increase their wellbeing and feel like they have made a difference [6]. Recruitment strategies that emphasise collective impact and sustainability may resonate strongly with younger demographics, potentially increasing participation in ecology-related citizen science.

Cluster analysis was explored to identify potential typologies of participant motivations, but poor separation between clusters suggests that these patterns are not strongly distinct. This reinforces the complexity of volunteer engagement and indicates that motivations may overlap rather than form clear categories. A limitation of this study is that we captured participants’ motivations only at the point of sign-up, and not throughout the project. Unfortunately, our attempts to gather feedback during the project were hindered by a low survey response rate. Citizen science projects could be strengthened by embedding the collection of information on participants’ ongoing motivations into the survey protocol, alongside other outcomes such as learning and changes in environmental behaviour.

The return rate of 12.75% may appear low but is comparable to the 10% return rate of the OPAL citizen science surveys, which also included a soil and earthworm survey [47]. However, other projects have achieved much higher return rates: the *Tea Bag Index – UK* reported 39% [48], the *Science Solstice* and *Summer Soil-Stice* projects assessing drug resistance in *Aspergillus fumigatus* achieved 75% [49] and *GenePools*, an eDNA project on pond biodiversity [50], had return rates between 70 - 93% (unpublished data).

One possible reason for this disparity was the lack of an immediate ‘reward’ for submitting results in *Earthworm Watch*. It is likely that additional participants completed the survey but did not upload their results. In contrast, the citizen science projects with higher return rates involved sample analyses that participants could not complete themselves, meaning that submitting data was necessary to receive full feedback or results. When return rates are critical to project success, future protocols could consider incorporating a reward element into data submission, tailored to participant motivations.

*Earthworm Watch* provided a valuable opportunity to explore not only the motivations of those who participated in a citizen science project, but also those who registered but did not take part. By examining motivations across age and gender and considering how these may relate to recruitment and survey completion, we offer insights into factors that may influence participation in citizen science. For future projects, tailoring recruitment strategies and feedback mechanisms to different demographic groups and motivational profiles may help enhance engagement and improve data return rates. For example, *Earthworm Watch* participants motivated by learning about and improving soil health in their gardens might have been more likely to submit data if they had received immediate, personalised interpretation of their results and advice based on them. Integrating career-focused content, such as interviews with practitioners and green careers advice through blogs and webinars, might encourage participation among those motivated by career aspirations. Acknowledging contributions through certificates or digital badges could further reinforce participant value and increase return rates.

## Conclusions

This study contributes to a growing body of research on participation in citizen science by examining both those who completed *Earthworm Watch* surveys and those who registered but did not take part. Despite the provision of physical materials, survey completion rates remained modest, with no significant difference between those who received packs and those who did not. This suggests that enabling immediate, low-barrier participation may be more effective than investing in physical resources. Additionally, while the recruitment route influenced engagement, only contact with project staff was associated with a significantly higher survey completion rate.

Our findings highlight the importance of understanding participant motivations - particularly the dominance of *understanding*- and *values*-based drivers - and how these vary across age and gender and influence the number of surveys submitted. We found that *social* motivations were more commonly reported by women, and that younger participants were more likely to cite *career*-related motivations.

These findings highlight the need for citizen science projects to adopt more tailored approaches to recruitment, design, and feedback - strategies that reflect the diverse motivations and demographic profiles of participants. For example, offering careers-related resources for those motivated by professional development. Such approaches can improve participation and data quality, ultimately enhancing both scientific and societal impact. This study also underscores the value of examining non-participation alongside participation, providing a more complete picture of engagement dynamics in citizen science.

## Electronic supplementary material

Below is the link to the electronic supplementary material.

Supplementary Material 1

## Data Availability

Data is available on the Natural History Museum Data Portal: Victoria J. Burton; Alan G. Jones; Lucy Robinson; Paul Eggleton; Andy Purvis (2025). Earthworm Watch Participants [Data set]. Natural History Museum. https://doi.org/10.5519/fbyflx0c.
