# Supplementary material for: Who watches the worms? Motivation and (non-)participation in a contributory citizen science project
Source: BMC Ecol Evol. 2025 Dec 24;26:10. doi: 10.1186/s12862-025-02471-y (PMC12849321; doi:10.1186/s12862-025-02471-y)
Supplement: Supplementary file 1 — Supplementary Material 1 [file 12862_2025_2471_MOESM1_ESM.docx]

# Who watches the worms? Motivation and (non-)participation in a contributory citizen science project

Supplementary Materials

Victoria J. Burton^a,b,*^, Alan G. Jones^c,1^, Lucy D. Robinson^a^, Paul Eggleton^a^, Andy Purvis^a,d^,

^a^ Natural History Museum, London, SW7 5BD, UK

^b^ Science and Solutions for a Changing Planet DTP, Department of Life Sciences, Imperial College London, SW7 2AZ, UK

^c^ Earthwatch, Mayfield House, 256 Banbury Road, Oxford, OX2 7DE, UK

^d^ Department of Life Sciences, Imperial College London, Silwood Park, SL5 7PY, UK

^*^ Corresponding author. E-mail address: [v.burton@nhm.ac.uk](mailto:v.burton@nhm.ac.uk)

^1^ Current address: Scion (New Zealand Forest Research Institute), Tı ̄tokorangi Drive, Private Bag 3020, Rotorua, 3046 New Zealand.


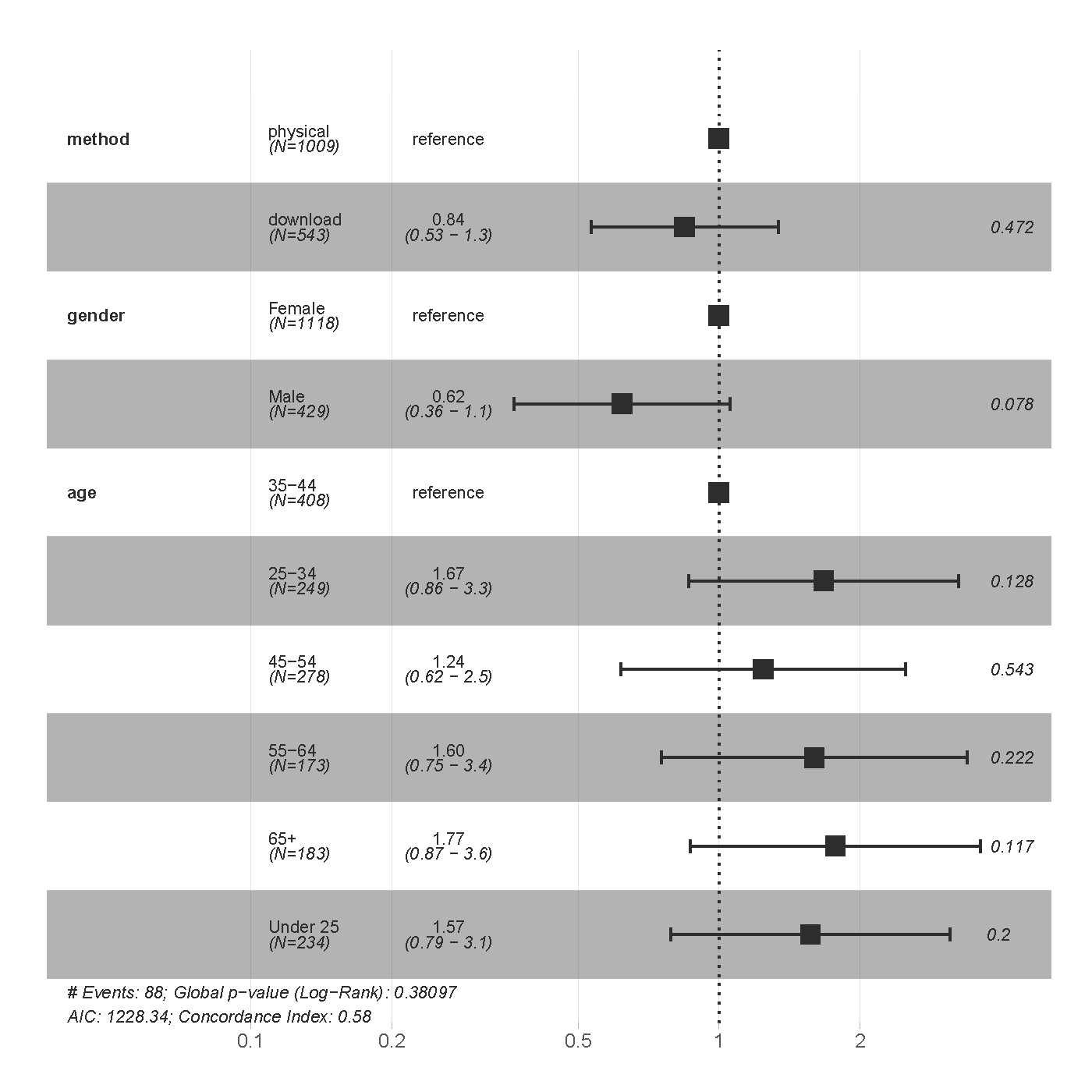


Figure S1. Summary of the Cox proportional hazards regression model for the likelihood of Earthworm Watch survey return. Hazard ratios (HR) with 95% confidence intervals (CI) are shown for each covariate. An HR > 1 indicates an increased likelihood of survey return, while an HR < 1 indicates a decreased likelihood, compared to the reference categories: posted pack, female gender, and age 35 - 44. The vertical line at HR = 1 represents no effect. N denotes the number of participants in each category. P-values are displayed on the right.


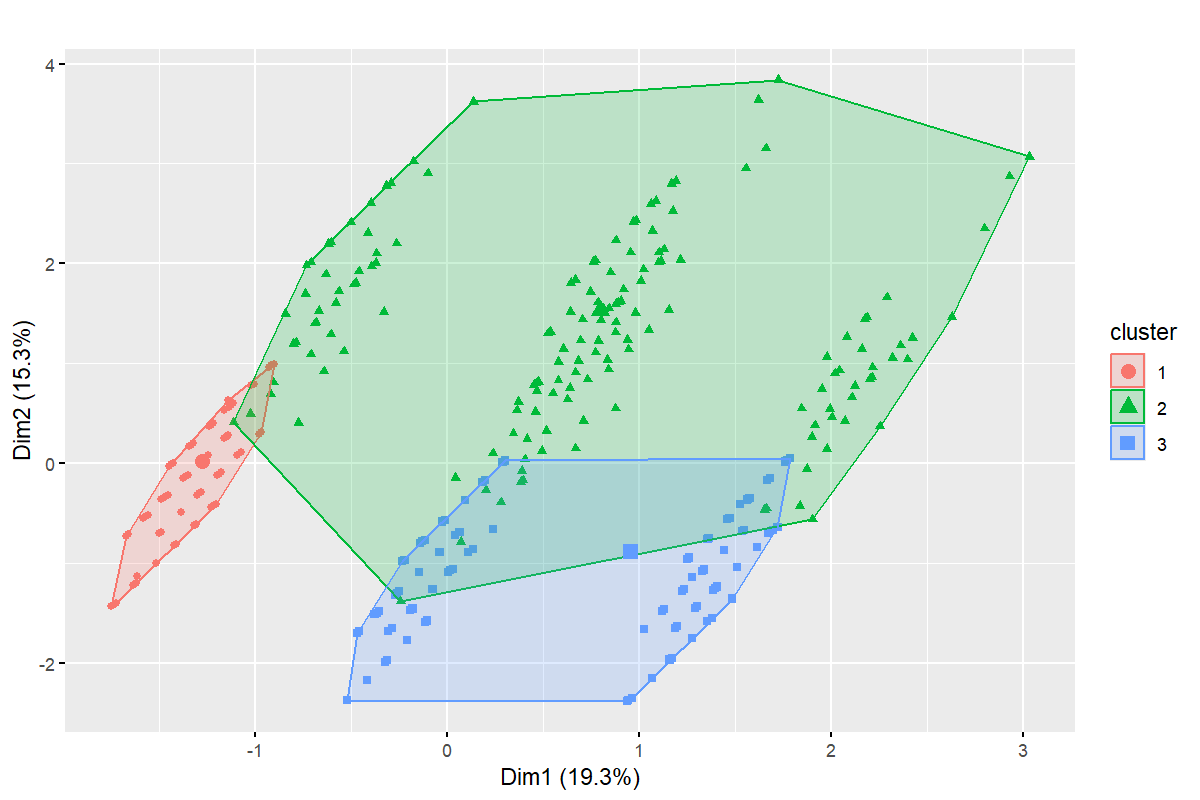


Figure S2. Cluster plot showing the grouping of Earthworm Watch participants into three clusters identified using hierarchical clustering with Ward’s method. Each point represents an individual participant, coloured by cluster membership. The plot illustrates the relative positioning of participants based on similarity in the variables used for clustering, with closer points indicating greater similarity.


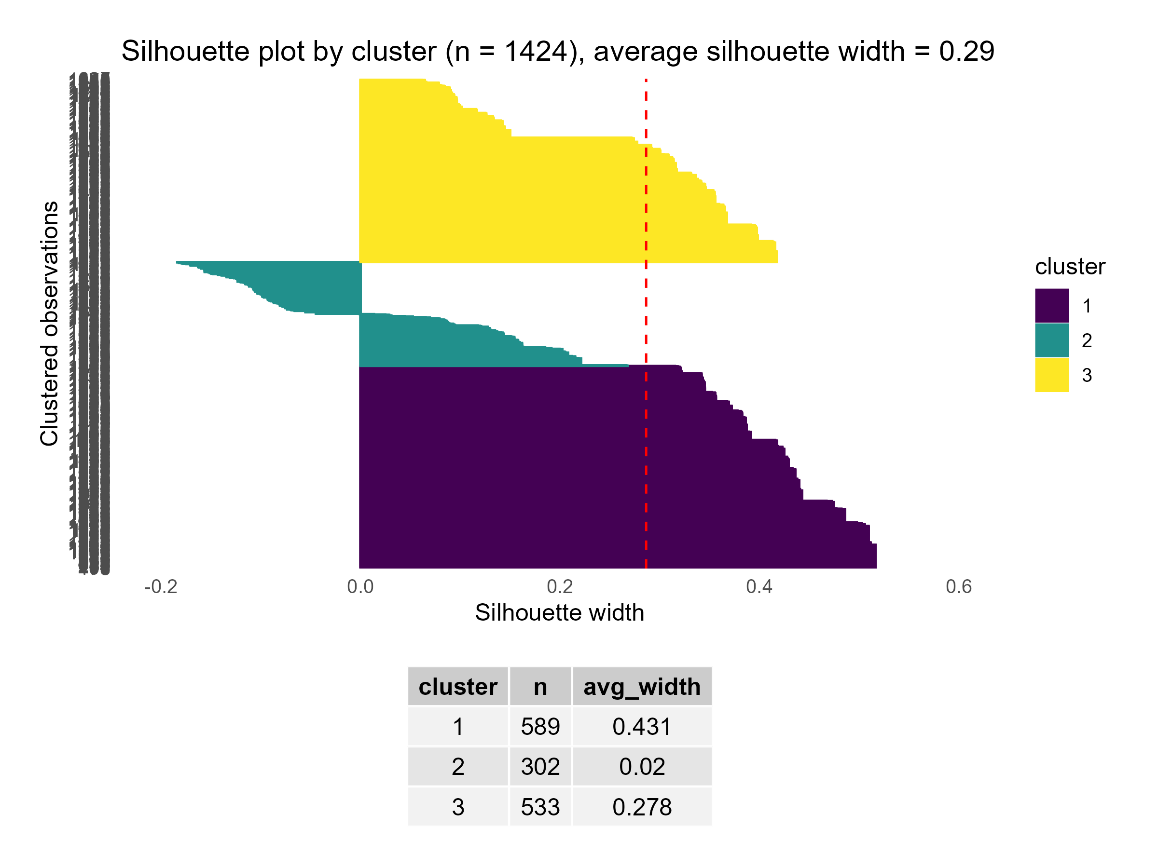


Figure S3. Silhouette plot for the three clusters of Earthworm Watch participants identified using hierarchical clustering with Ward’s method. The silhouette width indicates how well each participant fits within its assigned cluster (values closer to 1 suggest better cohesion and separation). The accompanying table summarises the number of participants in each cluster and the average silhouette width for each cluster, which reflects overall clustering quality.
